# Can invasions occur without change? A comparison of G-matrices and selection in the peach-potato aphid, Myzus persicae

**DOI:** 10.1002/ece3.883

**Published:** 2013-11-20

**Authors:** Leonardo D Bacigalupe, Karin Barrientos, Andrew P Beckerman, Mauricio J Carter, Christian C Figueroa, Stephen P Foster, Allen J Moore, Andrea X Silva, Roberto F Nespolo

**Affiliations:** 1 Instituto de Ciencias Ambientales y Evolutivas, Facultad de Ciencias, Universidad Austral de ChileP.O. 51110566, Valdivia, Chile; 2 Department of Animal and Plant Sciences, University of SheffieldSheffield, S102TN, U.K; 3 Centre for Ecology & Conservation, College of Life & Environmental Sciences, University of ExeterCornwall Campus, Penryn, U.K; 4 Instituto de Biología Vegetal y Biotecnología, Universidad de Talca2 Norte 685, Talca, Chile; 5 Rothamsted ResearchWest Common, Harpenden, Hertfordshire, AL5 2JQ, U.K; 6 Department of Genetics, University of GeorgiaAthens, GA, 30602

**Keywords:** Agriculture, aphids, **G**-matrix, invasive species, pest

## Abstract

Most evolutionary research on biological invasions has focused on changes seen between the native and invaded range for a particular species. However, it is likely that species that live in human-modified habitats in their native range might have evolved specific adaptations to those environments, which increase the likelihood of establishment and spread in similar human-altered environments. From a quantitative genetic perspective, this hypothesis suggests that both native and introduced populations should reside at or near the same adaptive peak. Therefore, we should observe no overall changes in the **G** (genetic variance–covariance) matrices between native and introduced ranges, and stabilizing selection on fitness-related traits in all populations. We tested these predictions comparing three populations of the worldwide pest *Myzus persicae* from the Middle East (native range) and the UK and Chile (separately introduced ranges). In general, our results provide mixed support for this idea, but further comparisons of other species are needed. In particular, we found that there has been some limited evolution in the studied traits, with the Middle East population differing from the UK and Chilean populations. This was reflected in the structure of the **G**-matrices, in which Chile differed from both UK and Middle East populations. Furthermore, the amount of genetic variation was massively reduced in Chile in comparison with UK and Middle East populations. Finally, we found no detectable selection on any trait in the three populations, but clones from the introduced ranges started to reproduce later, were smaller, had smaller offspring, and had lower reproductive fitness than clones from the native range.

## Introduction

Biodiversity on Earth is undergoing unprecedented transformation as the result of the effects of human activities on all kinds of ecosystems (Vitousek 1992; Mooney and Cleland 2001). Although land change use still is the main driver of biodiversity loss and habitat fragmentation (Hoffmann and Sgrò 2011), biological invasions represent the driver with most far-reaching consequences. Invasive species have caused more species extinctions than any other component of global change (Vitousek et al. 1997; Lockwood et al. 2007), have had a massive impact across every possible level in the biological hierarchy (Lee 2002; Ness and Bronstein 2004; Olden et al. 2004; Phillips and Shine 2006), and have induced evolutionary changes in native species as well as themselves evolving at an accelerated rate (Palumbi 2001; Phillips et al. 2006; Strayer et al. 2006). While the negative impact of invasive species has led to scrutiny by ecologists and conservation scientists, the evolutionary consequences of invasions are less often studied (Huey et al. 2005; Lockwood et al. 2007; Bacigalupe 2009). Given that the overall genetic characteristics of populations influence establishment and range expansion (Tsutsiu et al. 2000; Sakai et al. 2001; Lee 2002), an evolutionary perspective on invasions would offer invaluable insights into some of its mechanisms (Colautti et al. 2009). In particular, there is interest in understanding the role of the history of introduction, the genetic reshuffle at the founding process, and the genetic variation available to respond to natural selection in the introduced range (Lee et al. 2007; Bacigalupe 2009; Lee 2010; Ammunet et al. 2011; Calsbeek et al. 2011).

Any adaptive change in the mean phenotype from one generation to the next requires natural (or sexual) selection acting on heritable variation. Furthermore, when different traits share a genetic basis, summarized by genetic covariation, both the direction and rate of evolution can be facilitated or constrained (Schluter 1996; Steppan et al. 2002; Bégin and Roff 2003; Roff et al. 2004). The information on genetic variances and covariances for a suite of traits is contained in **G**, the additive genetic covariance matrix (Roff 2000) that shows the potentials and constraints to adaptive evolution in a particular system. Therefore, the comparison of **G-**matrices between the native and introduced range of a species has been proposed as critical to understand some of the evolutionary causes of invasion success (Bacigalupe 2009; Calsbeek et al. 2011).

Most evolutionary research on biological invasions has focused on changes seen between the native and invaded range for a particular species. However, it is likely that species that live in human-modified habitats in their native range might have evolved specific adaptations to those environments, which increase the likelihood of establishment and spread in similar human-altered environments in the new range. Although to the best of our knowledge direct tests of this idea are lacking, good candidate systems to evaluate it are crop pests.

Aphids (Hemiptera: Aphididae) are among the most successful herbivorous pest insects (van Emden and Harrington 2007; Foster et al. 2007), and the peach-potato aphid *Myzus persicae* is recognized as one of the most important agricultural pests in the world (Foster et al. 2007). This species feeds on over 400 plant species from 50 different families (Schoonhoven et al. 2005) and causes damage through direct feeding and/or transmission of plant viruses (van Emden and Harrington 2007). In addition, *M. persicae* shows different reproductive strategies, from obligate to cyclical parthenogenesis (i.e., alternating sexual with parthenogenetic reproduction), associated with the presence of its primary host, the peach *Prunus persica* (Simon et al. 2002). *Myzus persicae* is thought to have the same Eurasian origin as its primary host (Bortiri et al. 2001). In particular, several lines of evidence support the idea of a Chinese origin for the genus and that China was the place where peach was first cultivated (Huang et al. 2008). From China, peach cultivation moved westward to Persia (presently Iran) within the second to first century BC, and from there it spread to Europe and the Americas (Bassi and Monet 2008).

From a quantitative genetic perspective, the idea that species that live in human-modified habitats in their native range might have evolved specific adaptations to those environments leads to three predictions that can be tested by examining selection and genetic architecture. In both ranges, populations should have reached a similar point in the adaptive landscape, and it follows that first, we should observe no changes in overall genetic architecture when comparing populations from the native and introduced ranges. Second, the genetic architecture should not differ structurally. Finally, phenotypic means should not differ between the native and introduced ranges if populations are at a similar adaptive peak with stabilizing selection operating on traits associated with invasion success.

We tested those predictions using *M. persicae* collected from Turkey in the Middle East (native range; ME), the UK (introduced range), and Chile, where *M. persicae* was introduced less than 100 years ago, probably in the central part of the country (Fuentes-Contreras et al. 2004). We have no information regarding the history of *M. persicae* introduction to UK, but it is reasonable to suppose it occurred after peach was transported and cultivated in Persia (see above). Thus, we assume that the ME genotypes belonged to the native and both UK and Chile are “invaded” ranges.

## Materials and Methods

### Collection sites, maintenance, and microsatellite genotyping

Detailed information regarding sampling locations, maintenance conditions, and microsatellite genotyping for Chilean *M. persicae* can be found elsewhere (Castañeda et al. 2011). Briefly, 94 individual aphids were sampled next to roads and agricultural fields along an 1830-km latitudinal transect (27°S–41°S latitude) in Chile. In particular, aphids were collected in the northern and southern distributional limits as well as in places near the central parts of the distribution, yielding three different geographic zones (i.e., north, central, and south). Parthenogenetic colonies (aphid lineages) were established in small box cages (Blackman boxes) containing seedlings of *Capsicum annuum* var. *grossum* (cv. resistant). Each lineage was established from a single adult parthenogenetic wingless female. Aphid lineages were reared at 20 ± 1°C and LD 16:8 to ensure parthenogenetic reproduction, by transferring five wingless adults to new seven-day-old pepper seedlings every 10 days. Aphid colonies were reared on pepper seedlings for at least 10 generations before the experiments were done.

Each aphid lineage was genotyped using six previously described microsatellite loci (*Myz2, Myz3, Myz25, M35, M37,* and *M40*) (references in Castañeda et al. 2011). Forty-four (out of 94) redundant genotypes (i.e., colonies from the same genotype repeated more than once) were discarded. Most redundant genotypes were sampled from closed collection points, and only two aphid genotypes presented a widespread distribution according to the sampling transect (Castañeda et al. 2011). Thirty of those 50 genotypes were randomly selected for this study. In Europe, aphids were isolated from samples taken from a range of field and glasshouse crops in the UK and Turkey (Fenton et al. 2010). In each sample, aphids were collected, along with their supporting leaves, from plants at scattered positions throughout the collection site. Details on DNA extraction and microsatellite loci amplification for European genotypes can be found in Fenton et al. (2010). Fourteen genotypes were obtained from the UK and nine from the Middle East (ME) populations. Maintenance conditions for UK and ME aphids were the same as for Chilean clones.

### Breeding design

General husbandry for all clones (from Chile, the UK, and the ME) followed Castañeda et al. (2011). From each clonal genotype, ten parental wingless individual aphids were transferred to ten adult sweet pepper plants, collecting one nymph per plant after 24–48 h. This nymph (F1) was transferred to a Blackman box with 2-month-old pepper seedlings until maturity. Parental aphids were removed from seedlings after three days of parthenogenetic reproduction, and all but one nymph (F2) were discarded two days later. This process was repeated twice; hence, focal individuals were from the F4 generation. All aphids were maintained at 20 ± 1°C and LD 16:8.

### Phenotypes – reproductive fitness and morphological traits

The intrinsic rate of natural increase (*r*_*m*_) was determined according to Wyatt and White (1977). We determined the age of first reproduction (AFR) and the offspring number (ON) produced in an equal time. For example, if an aphid had its first nymph 7 days after being born (i.e., AFR), we counted its progeny for 7 days (i.e., ON). Then, *r*_*m*_ was calculated as *r*_*m*_ = 0.74·(log_e_ ON)/AFR, where 0.74 is a correction factor.

Size at birth was determined in 3–5 offspring individuals of the F4 generation. Each offspring was put on their back on a glass slide, and a digital picture of the right hind tibia was captured at 40X magnification (Olympus SZ6145TR Optics). Tibia lengths were obtained using the public software Image-J (http://rsb.info.nih.gov/ij/). The average of those 3–5 offspring was used in all statistical analyses. Adult body mass was determined on a MXA5 microbalance (Radwag, Czech Republic). Aphids were cooled on ice for a few seconds and weighed to the nearest microgram.

### Statistical analyses

For all analyses, traits were standardized (mean = 0, variance = 1). Family means among regions (i.e., Chile vs. UK vs. ME) were compared using a general linear model. Multiple comparisons of means were carried out with Tukey's contrasts, and results are presented as difference in standard deviations ± SE. As the Chilean data contain only two aphid genotypes with a widespread distribution (see Castañeda et al. 2011 for details), we also compared Chilean family means among the three geographic regions encompassed by the sampling transect (i.e., north vs. central vs. south).

We estimated and compared genetic covariance matrices using Bayesian Monte Carlo Markov chain mixed modeling approach (Hadfield 2010) combined with new tools for comparing covariance matrices and estimating covariance change along gradients (Robinson and Beckerman 2013). We used uninformative, parameter-expanded priors and collected 1000 samples of the joint posterior from 100,000 iterations after a burn-in interval of 500,000. Posterior modes (i.e., parameter estimates) and their 95% credible intervals were based on sampling 1000 times the posterior parameter distribution. Genotypes associated with specific zones were included as random effects. We fit one model to each location, resulting in three **G**-matrices.

We used two methods to compare G-matrices, and details on how they are implemented can be found in Robinson and Beckerman (2013). First, we compared pairs of G-matrices from each location. Specifically, we used a set of derived matrix comparison statistics, based largely around the eigen-decomposition (ordination) of **G** (see, e.g., Kirkpatrick 2009). This approach centers on evaluating differences in the variance–covariance relationship among traits between the two populations. The new methods, described in Robinson and Beckerman (2013), are based on using Bayesian MCMC model estimates of G-matrices to generate the derived metrics. The metrics, all well established in the literature, benefit from the Bayesian MCMC approach by having confidence intervals allowing for easy and strong inference.

Pairwise differences in variance and covariances reflect quantitative genetic hypotheses about the underlying genetic control of traits in the different populations, for example, environmental sensitivity of alleles, environment-specific loci, and pleiotropy. The metrics used in our analysis include the newly derived Ovaskainens “D” metric (Ovaskainen et al. 2008), describing differences in the underlying probability distribution of two G-matrices, to more historical metrics such as a comparison of variance associated with Gmax (major axis; Schluter 1996), of the overall magnitude of genetic variance, as well as perspectives on changes in covariance provided by Krzanowski's test (Krzanowski 1979) of the angles between the major axis of genetic variation (Gmax). As noted above, these derived metrics are all estimated in the Bayesian MCMC framework, which allow comparisons between **G**-matrices to be made with strong inference based on higher posterior density credible intervals (see Robinson and Beckerman 2013).

Second, it is possible to extend the pairwise comparison method to a method of tensors (Hine et al. 2009), which allows the analysis of G-matrices along an environmental gradient. We used this second method to analyze the change between Chile, the UK, and the ME, treating this geographic pattern as a gradient of invasion. The tensor-based approach, again executed in the MCMCglmm framework (see Robinson and Beckerman 2013), allows one to make inference about how and which traits contribute to structural change in genetic variance–covariance patterns across the gradient as a whole. The approach estimates the number of independent axes of multivariate genetic variance across an environmental gradient, estimates the genetic variance associated with these axes of multivariate genetic variance across the environmental gradient, and finally identifies which traits contribute toward multivariate changes across the gradient (see Hine et al. 2009; Robinson and Beckerman 2013).

Phenotypic selection analysis was only carried out in body mass and size at birth, as age of first reproduction is used to estimate individual *r*_*m*_. Given our relatively small sample size for the UK and the ME, we decided to evaluate the most parsimonious models of all potential ones, basically assessing whether populations have reached the adaptive peak (stabilizing selection) or not (directional selection). Thus, linear (β) and quadratic (γ) selection gradients were estimated by a forward stepwise regression model selection. Terms were added to the final model if they significantly contributed to a reduction in residual variation using *F*-tests. All analyses were carried out using R 2.15.0 (R Development Core Team. 2012).

## Results

### Phenotypic change in the New World: Means analyses in Chile

**Figure 1:**
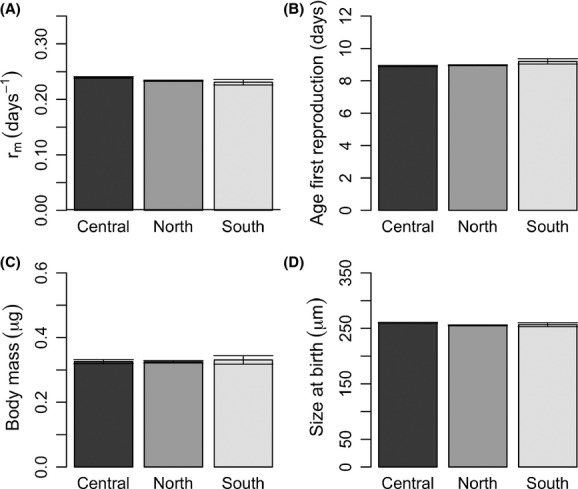
Phenotypic means for reproductive fitness (A), age of first reproduction (B), body mass (C) and size at birth (D) for the three regions of Chile where *Myzus persicae* was collected. *rm* is reproductive fitness. Data are presented as means ± 1 SE. Sample size for clonal families was as follows: central = 11, north = 15, south = 4.

We found no statistically significant difference among means among regions within Chile for any trait (AFR: *F*_2,27_ = 0.579, *P* = 0.567; *r*_*m*_: *F*_2,27_ = 0.588, *P* = 0.562; BM: *F*_2,27_ = 0.015, *P* = 0.985; SB: *F*_2,27_ = 0.502, *P* = 0.611), therefore suggesting that there is no evidence for a latitudinal cline (Fig. 1). Furthermore, for all traits the variance components associated with zone were not different from zero (results not shown), which is in agreement with the overall very low genetic variance we found for the country (see below). Therefore, for all subsequent analyses, we pooled all clone families from Chile.

### Phenotypic change across continents

**Figure 2:**
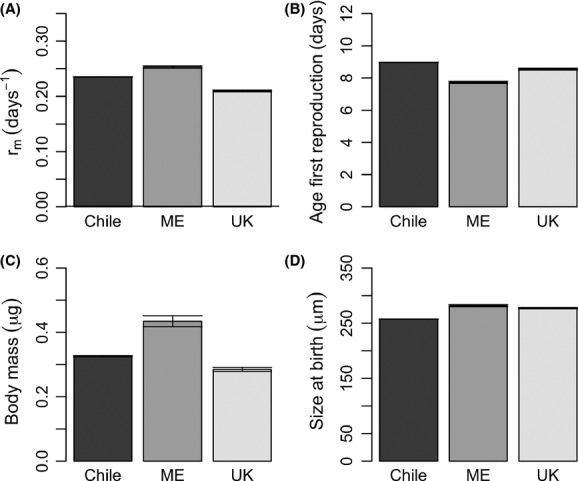
Phenotypic means for reproductive fitness (A), age of first reproduction (B), body mass (C) and size at birth (D) for Chile, United Kingdom (UK), and Middle East (ME). Data are presented as means ± 1SE. Sample size for clonal families was as follows: Chile = 30, UK = 14, ME = 9.

Reproductive fitness was different across all comparisons, with the UK clones having lower *r*_*m*_ values than both the ME (difference in standard deviations ± SE: −1.26 ± 0.24, *t*_50_ = −5.21, *P* < 0.001) and Chile (−0.74 ± 0.18, *t*_50_ = −4.04, *P* < 0.001), and marginally lower in Chile than in ME (−0.52 ± 0.21, *t*_50_ = −2.42, *P* = 0.049). As age of first reproduction is negatively correlated with individual *r*_*m*_, then the ME clones matured earlier (Chile–ME = 1.17 ± 0.20, *t*_50_ = 5.72, *P* < 0.001; UK–ME = 0.77 ± 0.23, *t*_50_ = 3.38, *P* = 0.004), but there was no significant difference between the UK and Chilean clones (UK–Chile = −0.39 ± 0.17, *t*_50_ = −2.26, *P* = 0.070). The ME clones were larger than both the UK and Chilean clones (UK–ME = −0.97 ± 0.24, *t*_50_ = −3.98, *P* = 0.001, Chile–ME = −0.70 ± 0.22, *t*_50_ = −3.24, *P* = 0.006), while there is no evidence to suggest that the UK and Chilean clones differed significantly (UK–Chile = −0.27 ± 0.18, *t*_50_ = −1.44, *P* = 0.324). Finally, the average size at birth was smaller in the Chilean clones (Chile–ME = −1.00 ± 0.21, *t*_50_ = −4.80, *P* < 0.001; UK–Chile = 0.82 ± 0.18, *t*_50_ = 4.61, *P* < 0.001), but there was no significant difference between the clones from UK and the ME (UK–ME = −0.18 ± 0.23, *t*_50_ = −0.78, *P* = 0.713). Overall, the ME clones started to reproduce earlier, got bigger, had bigger offspring, and had higher reproductive fitness than both the UK and Chilean clones (Fig. 2).

### Matrices estimation and comparison

Genetic variances and covariances are presented in Table 1 with broad-sense heritabilities and genetic correlations given in Table 2. In general, genetic variances were small for the three *M. persicae* populations, while heritability estimates were low to moderate (mean = 0.35, range = 0.09–0.66), with a clear pattern that follows the time since the original colonization: Mean heritability was 0.56 in ME, 0.35 in UK, and 0.13 in Chile. Genetic covariances were small, which resulted in low correlation estimates with wide credible intervals that included zero in all cases. It is likely that these results reflect low statistical power due to the small number of clonal families used (i.e., Chile = 30, UK = 14, ME = 9).

**Table 1:** Genetic variances (diagonal) and covariances (off-diagonal) for life history traits of *Myzus persicae* from Chile, United Kingdom (UK) and Middle East (ME).

Chile	AFR	BM	SB
AFR	0.13		
BM	0.0004	0.04	
SB	0.02	0.01	0.13

UK	AFR	BM	SB
AFR	0.43		
BM	−0.18	0.20	
SB	0.05	0.0007	0.28

ME	AFR	BM	SB
AFR	0.15		
BM	0.12	0.82	
SB	0.02	0.03	0.43

AFR, age of first reproduction; BM, body mass; and SB, size at birth.

**Table 2:** Broad-sense heritabilities (diagonal) and genetic correlations (off-diagonal) for life history traits of *Myzus persicae* from Chile, United Kingdom (UK), and Middle East (ME). 95% credible intervals are shown in parentheses.

Chile	AFR	BM	SB
AFR	0.14 [0.05, 0.27]		
BM	0.22 [−0.58, 0.65]	0.10 [5.4e−07, 0.20]	
SB	0.05 [−0.30, 0.62]	0.06 [−0.40, 0.73]	0.17 [0.06, 0.30]

UK	AFR	BM	SB
AFR	0.68 [0.42, 0.83]		
BM	−0.49 [−0.88, 0.05]	0.19 [0.04, 0.49]	
SB	0.15 [−0.53, 0.61]	−0.07 [−0.70, 0.53]	0.27 [0.07, 0.59]

ME	AFR	BM	SB
AFR	0.50 [0.25, 0.86]		
BM	0.22 [−0.48, 0.74]	0.56 [0.28, 0.86]	
SB	0.19 [−0.41, 0.84]	0.42 [−0.53, 0.73]	0.50 [0.21, 0.83]

AFR, age of first reproduction; BM, body mass; and SB, size at birth.

**Figure 3:**
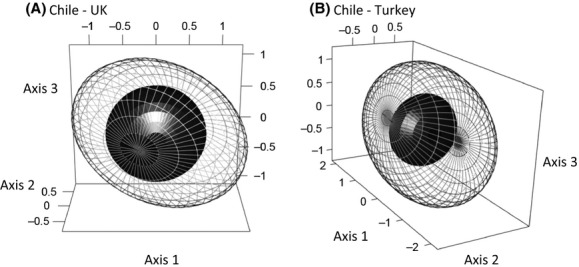
Subspace representation of the genetic variance–covariance in pairwise 686 comparisons between (A) Chile–UK and (B) Chile–Middle East. The hulls are an 687 ordinated (PCA) representation of the **G**-matrix. In both panels, the black hull captures the genetic variance–covariance in Chile, and the white hull the UK (A) or Middle East (B) genetic variance–covariance. The size of the hull is related to estimates of total genetic variance (Table 3), while the rotation of the hulls is related to the angles separating the major axes of genetic variance (Table 3 Gmax).

We found a significant Ovaskainen D between Chile and UK, which indicates that, in overall, matrices were different (Table 3). However, this is the only indication of difference between these two estimates of genetic covariance. Note that there appears to be a substantial volume reduction from UK to Chile (Fig. 3A), but there is no support for this being a significant difference (Table 3). The Chile–ME comparison also shows a significant Ovaskainen D, which is also supported by a major reduction in total genetic variance between the two populations (Table 3, Fig. 3B). Note also that although there is a rotation in the major axis visible in Fig. 3B, there is no statistical support for it (Table 3). Finally, we found no difference in any statistic for the UK–ME comparison (results not shown).

**Table 3:** Results of comparing the variance–covariance matrices for Chile with the UK and with the Middle East matrices. Ovaskainen D is an estimate of differences in the underlying probability distribution for two given G-matrices. Gmax corresponds to the major axis of phenotypic variation in each P. The Angle Between Gmax follows Krzanowski (1979). Sum Volume is a method for estimating total phenotypic variance. With the exception of Ovaskainen D, all derived metrics are presented as the difference in each metric between the two localities being compared. Bold rows are significant. For details on estimating the metrics, determining significance, and further definitions, see Robinson and Beckerman 2013.

	Chile–UK	Chile–ME
Ovaskainen D	**0.80**	**0.76**
Δ Variance Gmax	−0.08	−0.05
Angle Between Gmax	49.86	71.17
Δ sum-Volume	−0.91	−**1.78**

The tensor analysis (Table 4) revealed several complementary results augmenting the above insights. First, there is essentially only one major axis of genetic variation along the gradient. The first tensor captured 98% of the genetic variance across the gradient (CI: 0.61–0.99) with the second tensor accounting for the other 2% (CI: 0.0003–0.38). Second, along this gradient, the trait in which variance is most substantially reduced is size at birth (see Table 4, ΔVa). This complements what can be seen in the heritability estimates where the H^2^ for size at birth is lowest for this trait. Third, the genetic variance associated with each tensor is, as revealed in the matrix comparisons, highest in the ME site (Table 4; genetic variance of each tensor). Overall, these results suggest that: (i) there is a decreasing amount of genetic variance on which selection can act moving from ME to Chile, (ii) there is structural change along this gradient as revealed by differences in the underlying probability distribution characterizing the **G**-matrices, and (iii) these changes are largely tied to changes in size at birth.

**Table 4:** Tensor analysis output. (A) Genetic variance (mode, lower CI, upper CI) associated with each tensor describing variance–covariance change across the gradient. (B) Directional change in genetic variance for each trait (ΔVa) associated with Tensor 1, which explains 98% of the genetic variance across the gradient. ± symbols denote relative changes. (C) Genetic variance at sites associated with Tensor 1.

	Mode	Lower	Upper
(A) Genetic variance in each tensor			
Tensor 1	0.98	0.61	0.9996262
Tensor 2	0.021	0.00037	0.3852562
(B) Δ Va Tensor 1			
Age first reproduction	0.015	−0.103	0.99
Size at maturity	−0.022	−0.588	0.35
Size at birth	−0.98	−0.99	−0.027
(C) Genetic variance at sites associated with T1			
Chile	0.073	0.0029	0.201
UK	0.17	0.00039	1.55
Turkey	1.55	0.012	4.93

### Phenotypic selection

For Chile and the UK, there was no evidence for directional or stabilizing selection on any trait (all *P* > 0.05), which suggests that all phenotypic variation for these traits in the introduced regions is at, or practically at, the optimum. For the Middle East, the best-supported model was the one that included directional selection on size at birth (estimate = −0.044, SE = 0.014, *F*_1,65_ = 10.05, *P* = 0.002).

## Discussion

We evaluated the hypothesis that species that live in human-modified habitats in their native range might have evolved specific adaptations to such environments, which increase the likelihood for establishment and spread in similar altered environments in the new range. If true, under a quantitative genetic framework we should observe no overall changes in the structure of **G** between native and introduced ranges, and we should observe stabilizing selection on fitness-related traits, as populations should be at a similar adaptive peak. In general, our results provide mixed support for this idea, but further comparisons of other species are needed. In particular, we found that there has been some limited evolution in the studied traits, with the Middle East population differing from the UK and Chilean populations. This was not supported by our comparison of the structure of the **G**-matrices, which was different between Chilean and both UK and ME populations. Furthermore, the amount of genetic variation was massively reduced in Chile in comparison with UK and ME. Finally, we found no detectable selection on any trait in the three populations, but clones from the introduced ranges started to reproduce later, were smaller, had smaller offspring, and had lower reproductive fitness than clones from the native range (i.e., ME). Thus, it may be that our hypothesis should be more nuanced regarding change.

### Phenotypic means within Chile: No evidence for population subdivision

Evolutionary inferences can be blurred when latitude is a confounding factor (Bacigalupe 2009; Colautti et al. 2009). To exclude evolutionary change and therefore population subdivision following the introduction and spread of *M. persicae* through the country, we compared the means of the 4 fitness-related traits among the three geographic zones in Chile, which encompass the whole distributional range of the species. We found no information to suggest a genetically based latitudinal cline in any of the studied traits. Interestingly, these results support aspects of the hypothesis at a local scale, namely that populations are at a similar adaptive peak. In this context, it does not seem that rapid evolution within the introduced range was required for the invasion success of this species. Adaptive evolution during the expansion phase depends on the current amount of genetic variation in founder populations and the relative role of other factors at the founding processes (e.g., genetic drift, multiple introductions). Although we lack molecular data, our results strongly suggest a strong founder effect, as the overall amount of genetic and phenotypic variation for all traits in Chile is extremely low in comparison with populations in the Old World (see below).

### Evolutionary change across regions and selection

We compared the phenotypic change between native and introduced ranges in aphids grown under common garden conditions for at least 10 generations to evaluate evolutionary change between the three populations. Given that all clones were reared under identical conditions, any persistent phenotypic differences among regions should reflect genetic differences among clones, which suggests an evolutionary change as a result of either selection or genetic drift (Kawecki and Ebert 2004). Our results suggest that all evaluated traits have evolved: In general, clones from the native range (ME) started to reproduce earlier, got heavier, had bigger offspring, and had higher reproductive fitness than clones in the introduced ranges (UK and Chile).

It is difficult to unambiguously determine the direction of evolutionary change in our populations, as there was not a clear pattern of change in the means of the studied traits. Furthermore, there are several selective factors that might vary either temporally or spatially (e.g., host plants, natural enemies, insecticides, mode of reproduction) and thus that might be responsible for genotypic selection in the field (e.g., Zamoum et al. 2005; Vorburger 2006; Margaritopoulos et al. 2009) and ultimately on the observed population means. Unfortunately, we lack precise information for all those selective factors on the three regions we studied, but it seems likely that regions differ quite substantially in most, if not all, of them. Nevertheless, on the one hand, our results suggest that *M. persicae* has changed in the invaded regions, probably as a consequence of a strong founder effect. We detected no selection operating on any of the traits in either the UK or Chile, and we estimated a smaller amount of genetic variation (see below), particularly in Chile. However, our results also indicate that evolutionary change might be occurring in the native *M. persicae* range as we detected directional selection for smaller size at birth in the Middle East clones. The cause of that selection and why it differs from the introduced populations remains unclear.

We are aware that the location of the common garden (i.e., Chile in this study) may affect the phenotypic expression of the studied traits and thus the estimated differences between regions and their biological interpretation (Williams et al. 2008; Colautti et al. 2009). In particular, although *M. persicae* was grown in well-known standard conditions for the species (Silva et al. 2012), there might have been some genotype-specific responses (i.e., GxE) that might have influenced our results given that multiple garden locations were not used. Nonetheless, the pattern we detected is consistent enough to suggest that GxE might not be an issue. There was no evidence to suggest evolutionary change within Chile even when only two aphid genotypes were widespread. In spite of important differences between Chile and the UK (e.g., reproduction mode), we found several similarities at different levels between both regions with respect to the ME. Furthermore, if there is local adaptation, then the location of the common garden should favor local genotypes. Our results do not suggest that the Chilean clones were favored: For all traits studied, their phenotypic means were equal to or smaller than those of the native range clones.

Comparison of the genetic architecture complements well the previous findings and offers no strong support for the hypothesis evaluated; **G**-matrices between UK and ME do not differ statistically (although the UK population showed lower levels of variation than the ME population) while both have much more variation than Chile (Fig. 3). Thus, our results suggest that the Chilean populations may have suffered a strong bottleneck during the introduction and founding processes (see also Zepeda-Paulo et al. 2010). Therefore, mean changes across native and invaded ranges may be a consequence of the colonization process followed by asexual reproduction, and not necessarily a direct result of evolution itself in the new environment. Regardless of the measured levels of genetic variation, this species thrives to the point of being a huge agronomic and economic problem in both the UK and Chile as a consequence of direct feeding and/or transmission of plant viruses to crops. There are several reasons that may account for this. First, *M. persicae* show great phenotypic plasticity as it possesses a great diversity of enzymatic detoxification systems as a consequence of feeding upon many plant hosts (Mello and Silva-Filho 2002; Francis et al. 2006; Ramsey et al. 2010). Second, target site mutations that confer insecticide resistance and have probably evolved as a result of that management also play a role against highly defended hosts (Silva et al. 2012). Third, transgenerational induction mechanisms allow genotypes without insecticide resistance mutations to compensate on reproductive fitness when feeding on hosts with high levels of allelochemicals (L. D. Bacigalupe, A. X. Silva, C. C. Figueroa, S. P. Foster, A. P. Beckerman, and A. J. Moore, unpublished data, Holeski et al. 2012). As a pest, *M. persicae* is under strong human-induced selective pressures that have shaped the genetic architecture of its populations. Further evolution in this species might be associated with the decisions humans have to make on crop management (e.g., insecticide regimens, host rotation, reproductive mode, human transport and commerce, etc.) (Margaritopoulos et al. 2009). In this context, it is hard to imagine a species living in a human-modified habitat that it is not subject to similar management decisions. Thus, potential candidates to test the evaluated hypothesis might be species that have recently become a pest in the introduced ranges.
